# Microfluidic Enrichment and Computational Analysis of Rare Sequences from Mixed Genomic Samples for Metagenomic Mining

**DOI:** 10.1089/crispr.2022.0054

**Published:** 2022-10-13

**Authors:** Naiwen Cui, Guihem Faure, Ankita Singh, Rhiannon Macrae, Feng Zhang

**Affiliations:** ^1^ Howard Hughes Medical Institute, Cambridge, Massachusetts, USA; Massachusetts Institute of Technology, Cambridge, MA 02139, USA.; ^2^ Broad Institute of MIT and Harvard, Cambridge, Massachusetts, USA; Massachusetts Institute of Technology, Cambridge, MA 02139, USA.; ^3^ McGovern Institute for Brain Research, Massachusetts Institute of Technology, Cambridge, MA 02139, USA; Massachusetts Institute of Technology, Cambridge, MA 02139, USA.; ^4^ Department of Brain and Cognitive Science, Massachusetts Institute of Technology, Cambridge, MA 02139, USA; and Massachusetts Institute of Technology, Cambridge, MA 02139, USA.; ^5^ Department of Biological Engineering, Massachusetts Institute of Technology, Cambridge, MA 02139, USA.

## Abstract

Many powerful molecular biology tools have their origins in natural systems, including restriction modification enzymes and the CRISPR effectors, Cas9, Cas12, and Cas13. Heightened interest in these systems has led to mining of genomic and metagenomic data to identify new orthologs of these proteins, new types of CRISPR systems, and uncharacterized natural systems with novel mechanisms. To accelerate metagenomic mining, we developed a high-throughput, low-cost droplet microfluidic-based method for enrichment of rare sequences in a mixed starting population. Using a computational pipeline, we then searched in the enriched data for the presence of CRISPR-Cas systems, identifying a previously unknown CRISPR-Cas system. Our approach enables researchers to efficiently mine metagenomic samples for sequences of interest, greatly accelerating the search for nature's treasures.

## Introduction

Microbial CRISPR-Cas systems, which use an RNA guide to direct the effector protein or complex to a nucleic acid sequence, have been engineered for use as powerful programmable molecular technologies. There is a large diversity of CRISPR-Cas systems, many of which have distinct modes of action.^1^ For example, Class 1 systems use the Cascade complex to cleave target sequences, whereas Class 2 systems use a single effector protein, such as Cas9, to cleave target sequences. Within these classes, there is substantial mechanistic variation, and these unique characteristics form the basis for a range of genome and transcriptome editing tools as well as nucleic acid detection platforms.^2^

The full scope of this diversity continues to emerge through bioinformatic efforts to identify previously unknown CRISPR-Cas systems from genomic and metagenomic sequences.^3–6^ This type of approach has been extended to look for other types of natural systems, including other RNA-guided systems^7^ and novel microbial defense systems.^8,9^ However, these efforts are limited by the availability of the sequences, and although sequencing continues apace, it remains challenging and often prohibitively expensive to sequence rare genomes.^10,11^

To address this challenge, we developed a high-throughput, low-cost droplet microfluidic-based workflow for enrichment of sequences of interest in a mixed microbial population. We show that our method can achieve over 400,000-fold enrichment of a rare species in a complex sample, and we apply it to identify a previously unknown CRISPR-Cas system.

## Materials and Methods

### Microfluidic device fabrication

All microfluidic devices used in this article were fabricated using polydimethylsiloxane with the standard soft lithography method.^12^ Channels then underwent a hydrophobic surface treatment by flowing through Aquapel (PPG, Pittsburgh, PA), followed by flushing with pressured nitrogen. In the picoinjection devices, the metal alloy, Indalloy 19 (51 In, 32.5 Bi, and 16.5 Sn; 0.020 inch diameter), was used for electrode fabrication.

On one end of the electrodes, inlets were first blocked using eight-pin terminal blocks (Phoenix Contact, Middletown, PA). Devices were then preheated using a hot plate at 95°C for 5 min. Indalloy was then inserted and pushed into the other inlet; it then flows through the entire channel due to its low melting temperature until it reaches the pin terminal block. The hot plate was then turned off to allow electrodes to solidify. The double-layered device (double emulsion device) was fabricated using a previously published protocol,^13^ with a 30-μm-deep first layer and 50-μm-deep second layer.

Our microfluidic setup also contains a high-speed camera (HiSpec 1; Fastec Imaging), which allows us to examine droplet integrity during each step of our experiment.

### Bacterial sample preparation

For the *Staphylococcus aureus* enrichment test case, we used a Zymo bacterial sample kit (D6310), a premixed sample of microbes containing eight bacterial species (three Gram-negative and five Gram-positive) and two species of yeast. We verified the ratio of microbes by sequencing. This sample was washed three times by centrifugation and resuspended with 1 mL of phosphate-buffered saline (PBS).

Bacterial abundance was determined using a bacterial live/dead assay (L7012; Thermo Fisher Scientific) and hemacytometer (DHC-N21; Bulldog Bio) before proceeding to the encapsulation step. For the Antarctic sample (a gift from Roger Summons), a small portion of the freeze-dried sample was immersed in 1 mL of PBS in a 1.5-mL Eppendorf tube. The tube was then taped onto a vortex mixer and vortexed for 10 min at high speed to resuspend single bacteria.

The suspension was then filtered using a 40-μm strainer (352340; Falcon) to remove large debris and a 5-μm filter (SLSV025LS; Millex-SV) to remove any particles that could potentially clog the microfluidic channel (while allowing bacteria to pass through). The sample was then washed three times with centrifugation and resuspended in 1 mL of PBS to remove any free-floating DNA.

Finally, bacterial abundance was determined using a bacterial live/dead assay (L7012; Thermo Fisher Scientific) and hemacytometer (DHC-N21; Bulldog-Bio).

### Co-flow encapsulation of bacteria and lysis buffer

To encapsulate bacteria, we first prepared 50 μL of lysis buffer with the following composition: 10 μL of prepGEM green buffer (PBA0100; MicroGEM), 1 μL of lysozyme (PBA0100; MicroGEM), 1 μL of prepGEM (PBA0100; MicroGEM), 28.5 μL of water, 7.5 μL of random hexamer (100 μM, SO142; Thermo Fisher Scientific ), and 2 μL of Tween 20 (10%; P9416-50ML; Sigma).

We also prepared the bacterial phase: ∼3 million bacteria from the bacterial sample preparation were added to 21.25 μL of PBS and vortexed briefly to mix, then we added 4.75 μL of OptiPrep (D1556-250ML; Sigma) to the solution, followed by careful pipetting to mix and ensure that single bacteria remain resuspended during the entire encapsulation process. These two solutions were then placed in separate 1-mL syringes (BD-309628; VWR) that were preloaded with 300 μL of HFE-7500 oil (Novec 7500; 3M). Next, blunt needles (B27-50; SAI Infusion Technologies) and PE-2 tubing (BB31695-PE/2; Scientific Commodities Incorporated) were put on both syringes and then placed on syringe pumps (782910; KD Scientific).

Approximately 3 million monodispersed 30-μm diameter droplets were generated using a co-flow encapsulation device with a flow rate of 100 μL/h for the lysis buffer, 100 μL/h for the bacterial phase, and 400 μL/h for the HFE-7500 + 2% surfactant (008-FluoroSurfactant-5G; RAN Biotechnologies) phase. Drops were collected in 1.5-mL Eppendorf tubes and transferred to polymerase chain reaction (PCR) tubes, and then 100 μL of extra-heavy mineral oil (700000-456; VWR) was added on top of the emulsion to prevent evaporation during the PCR.

The collected drops were then thermocycled using the following protocol: 37°C for 15 min, 75°C for 10 min, 95°C for 5 min, and a hold step at 4°C. We confirmed microbe encapsulation by microscopy and verified that we have a single microbe in >60% of the drops that contain microbes, in line with our theoretical Poisson loading (distribution of 1).

### Injection of the multiple displacement amplification reagent

Mineral oil was carefully removed from the drops in the PCR tube and then drops were carefully pipetted and loaded into a syringe with 300 μL of preloaded HFE-7500 + 2% surfactant using a P-200 pipette. A PEEK adaptor (F-112 and P-662; IDEX Corporation) and PEEK tubing (TPK.510-100FT; Vici Precision Sampling) were attached to the syringe before loading the syringe on the syringe pump. A total of 100 μL of the multiple displacement amplification (MDA) reagent solution [2 × Phi29 DNA polymerase buffer (30221-2; Lucigen), 10 μM random hexamer, 0.5 mM deoxynucleoside triphosphate (dNTP) mix (18-427-088; Fisher Scientific), 0.1 × Phi29 polymerase (30221-2; Lucigen), 2.5 mg/mL Bovine Serum Albumin (BSA) (AM2618; Thermo Fisher Scientific), and 0.5% Tween 20] was then loaded into a syringe with 300 μL of preloaded HFE-7500.

A blunt needle and PE-2 tubing were then attached to the syringe before loading the syringe on the syringe pump. After priming, drops were reinjected into the picoinjection microfluidic device and separated by the oil +2% surfactant, with equal spacing between two adjacent drops. Upon arrival at the injection region, a 20-kHz sine wave, generated by a signal generator (AFG1000; Tektronix) and voltage amplifier (Model 2220; Trek), destabilizes the drop surface such that a fixed amount of MDA reagent can be injected into each droplet at ∼1400 Hz.

The flow rates used were as follows: oil +2% surfactant: 180 μL/h; reinjected drops: 100 μL/h; and MDA reagent: 75 μL/h. Drops were then collected and thermocycled using the following protocol: 4°C for 10 min, 30°C for 16 h, and a hold step at 4°C.

### Droplet splitting

Following whole-genome amplification, drops (∼30 μm) were reinjected into a droplet splitter device to separate each droplet into two equal-sized smaller droplets (∼24 μm) (flow rate 200 μL/h). The split drops were collected in separate PCR tubes. For one-half of the split drops, 100 μL of extra-heavy mineral oil was added on top, and drops were kept at 4°C for later use.

The split drops in the other half were then broken by adding 100 μL of 20% 1H,1H,2H,2H–perfluoro-1-octanol in HFE-7500, followed by brief centrifugation. The supernatant was collected using a P-20 pipette, and library construction was performed using the NEBNext Ultra II FS kit (E7805L), following the protocol for a 100-ng sample. The library was then sequenced on a NextSeq High-Output 75-bp cycle kit (Illumina) with the following exception: we performed a 91-bp cycle for Read 1 only and no index sequencing.

### De novo assembly

Sequencing reads were first filtered using Trimmomatic with a minimum length of 85 bp and CROP:90 bp.^14^ Genome assembly was performed using both MEGAHIT^15^ and SPAdes^16^ using their default settings. A taxonomy analysis and open reading frame (ORF) prediction were then performed using Contig Annotation Tool.^17^ The ORF-predicted contigs were then aligned against a CRISPR-associated protein database using hmmsearch.^18^ CRISPR arrays were predicted using MinCED with the default setting.^19^ A custom iPython code was written to collect all contigs with a CRISPR array, with/without CRISPR-associated proteins, and analyzed further.

Finally, selected reads were loaded in Geneious for downstream analysis using the following manual pipeline: predict ORF and CRISPR arrays, find the contig that contains the target region, and examine the new genes nearby using HHPred^20^ and BLAST.^21^

### Probe design

Probes for target contigs were designed using Bio-Rad's ddPCR design protocol (https://www.bio-rad.com/en-us/life-science/learning-center/introduction-to-digital-pcr/planning-ddpcr-experiments). For each target, a forward primer, reverse primer, and probe (250 nm PrimeTime 5′ 6-FAM/ZEN/3′ IBFQ, high performance liquid chromatography [HPLC] purified) were designed and then ordered from IDT.

### Injection of the droplet digital polymerase chain reaction reagent into drops

The drops stored at 4°C were used for droplet digital polymerase chain reaction (ddPCR) following the probe design. After removing the top mineral oil, drops were loaded into a syringe, as described above. The ddPCR reagent [2 × FastStart 10 × buffer without MgCl_2_ (12 032 902 001; Roche), 4 mM MgCl_2_ (12 032 902 001; Roche), 1.44 μM forward primer (IDT), 1.44 μM reverse primer (IDT), 0.4 μM probe (IDT), 1.2 μM deoxyuridine triphosphate (dUTP) (N0459S; NEB), 2.5 mg/mL BSA, 0.4% Tween 20, 0.8 μM dNTP (R1121; Thermo Fisher Scientific), and 0.16 U/μL polymerase (12 032 902 001; Roche)] was injected into each drop in the same way the MDA reagent was injected.

dUTP was used so that PCR products can be digested after sorting before the final whole-genome amplification step (see Section “Emulsion whole-genome amplification”). One hundred microliters of mineral oil was added to the top of the collected drops, and drops were then thermocycled using the following protocol: 95°C for 4 min, 40 cycles × (95°C for 30 s, 55°C for 30 s, and 72°C for 45 s), 72°C for 5 min, and a hold step at 4°C.

### Double emulsion and fluorescence-activated cell sorting

Drops were reemulsified in oil +2% Pluronic F-68 using a previously published protocol^22^ (cite Brower *et al.*) to achieve water-in-oil-in-water double emulsion drops. Double emulsion drops were collected into low-bind Eppendorf tubes (0030122348; Eppendorf) using a Sony SH800 FACS sorter with a 130-μm nozzle and ∼600 Hz sorting speed.^22^

### Emulsion whole-genome amplification

After sorting, Eppendorf tubes were spun at maximum speed (∼21,000 *g*) for 1 min. Tubes were then placed in a 37°C benchtop incubator with the lids open to allow the sorted drops to dry, thus breaking the double emulsion. After drying (∼1–2 min), 0.66 μL of DNase, RNase-free water, and 0.34 μL of USER enzyme (M5508S; NEB) were added to the tube, lids were closed, and tubes were vortexed for 10 s and then briefly centrifuged to collect the droplet.

This process was repeated three times for maximum recovery. Samples were then digested at 37°C for 15 min (by the USER enzyme) and heat inactivated at 65°C for 10 min. Whole-genome amplification was then performed following the REPLI-g protocol for single cell amplification of purified genome DNA for a total 20-μL reaction with 1.25 mg/mL BSA (150343; Qiagen). Sixteen microliters of HFE-7500 with 2% surfactant was added, and emulsions were generated by pipetting with a P-1000 pipette.

Drops were then transferred to a PCR tube using a P-200 pipette and thermocycled using the following protocol: 4°C for 5 min, 30°C for 4 h, and a hold step at 4°C. After amplification, emulsions were broken, the supernatant was collected as described above, and samples were sequenced using a Nextera XT kit (FC-131-1096; Illumina). The library was then sequenced on the MiSeq or NextSeq (Illumina) machine, following the manufacturer's protocol.

## Results

**FIG. 1.**
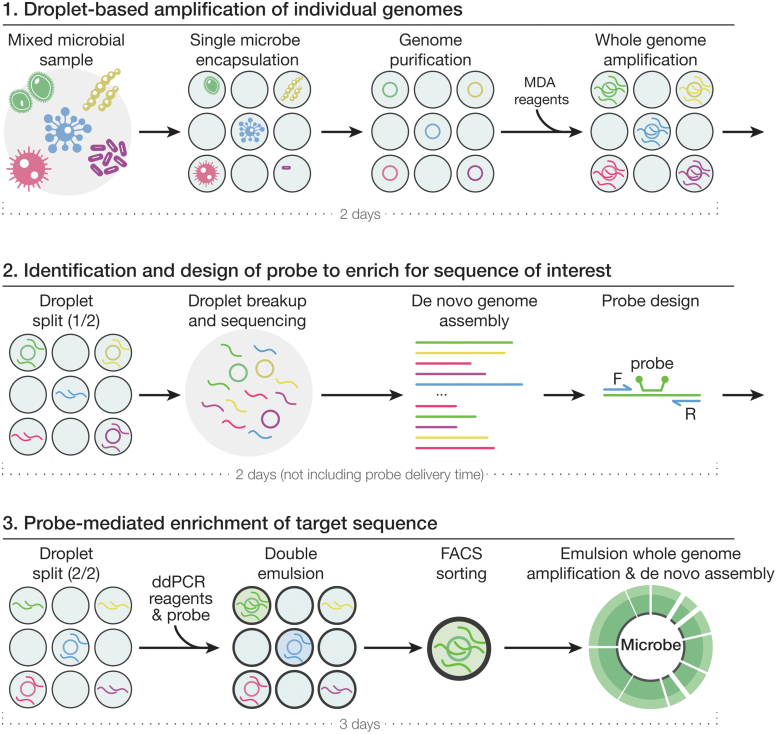
Workflow of the three-step enrichment method. In step 1, environmental microbes are encapsulated into picoliter water-in-oil droplets and lysed inside each droplet with a Poisson distribution of 1. The multiple displacement amplification reagent is then injected into each droplet with a picoinjection device and whole-genome amplification is performed overnight. Each droplet is split evenly into two smaller droplets, one of which is broken immediately for step 2, and the other of which is saved for step 3. In step 2, after droplet breakup, genomes are sequenced and contigs are assembled and searched for a sequence of interest. In step 3, droplet digital polymerase chain reaction reagents along with the probe set designed to match the sequence of interest are injected into the second droplet split using a picoinjection device. After double emulsion droplet generation, droplets are sorted by fluorescence-activated cell sorting to isolate those droplets that contain the sequence of interest. The double emulsion droplets are then broken, and emulsion whole-genome amplification is performed, followed by sequencing and *de novo* assembly.

Our method consists of three main steps (Fig. 1). In step 1, we encapsulate ∼1 microbe in picoliter droplets with the lysis buffer (see the Materials and Methods section for details). After lysis and heat inactivation, we add the MDA reagent to each droplet for whole-genome amplification.

In step 2, each droplet is split into two equal-sized smaller droplets, each collected in a separate tube. We break the droplets in the first split and sequence and assemble the DNA. Then, we computationally identify a region of interest that we want to enrich and design a ddPCR probe and primers for this region.

In step 3, we inject the probe along with ddPCR reagents into the second droplet split and perform ddPCR to amplify the target sequence, which results in higher fluorescence intensity for the droplets containing the target gene. We then reemulsify the droplets to form water–oil–water double emulsion droplets to enable fluorescence-activated cell sorting (FACS)-based enrichment. Finally, we generate emulsions using a P-1000 pipette and perform whole-genome amplification in sorted droplets, followed by sequencing and *de novo* assembly.

**FIG. 2.**
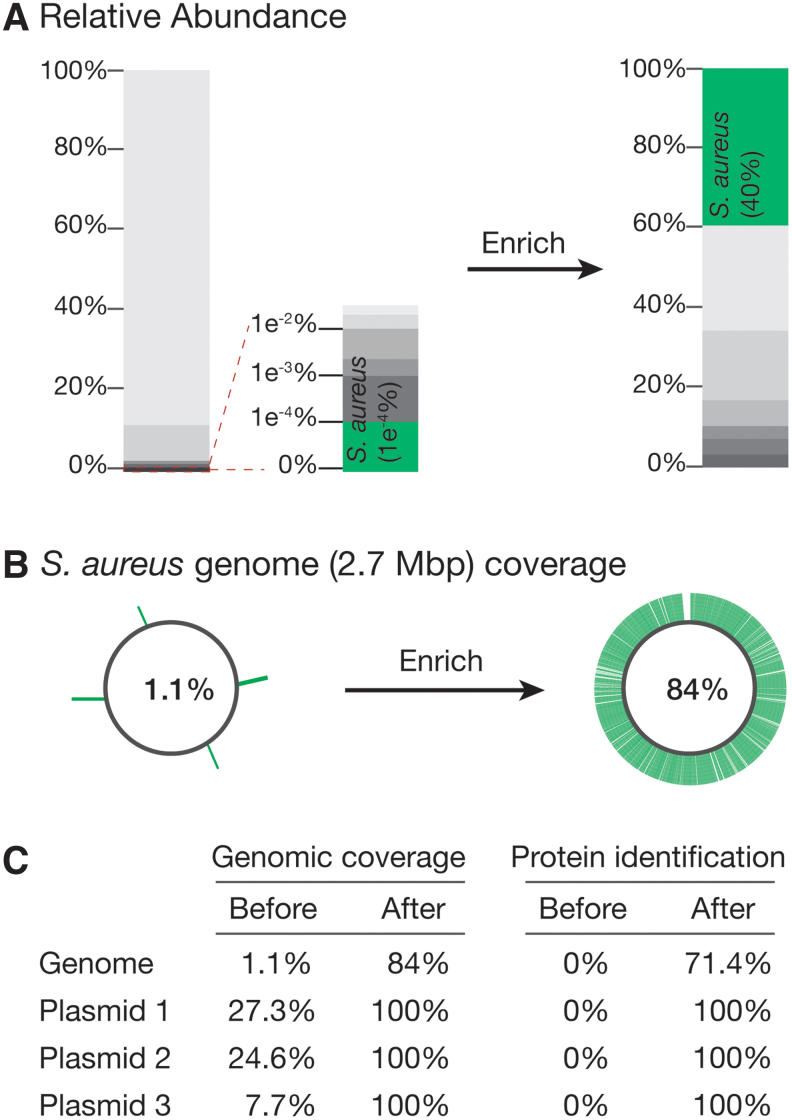
Enrichment of a low-abundance microbe in a log-distributed 10-microbial sample. **(A)** We applied our method to a log-distributed 10-microbial community standard and designed a probe targeting a 175-bp protein-coding region of *Staphylococcus aureus* (relative abundance ∼1 in 1,000,000 in the starting sample). After enrichment, *S. aureus* made up nearly 40% of the population. **(B)** We identified a 42,903-bp continuous DNA contig containing the target region and achieved 84% genome coverage. **(C)** We achieved 71.4% protein coverage across the entire genome as well as 100% genome and protein coverage on all three plasmids.

As an initial proof of concept of our method, we first sought to enrich a low-abundance bacterial species, *S. aureus*, from a defined mix of 10 microbes. In the initial sample, *S. aureus* was present at ∼1 in 1,000,000 (Fig. 2A). Traditionally, to encapsulate a single microbe in droplets, one would encapsulate with a Poisson distribution of 0.1, meaning the microbe number = 0.1*x* (where *x* is the total number of drops). This would result in 90% of the droplets being empty, and 10% of the droplets would contain microbes. Among those 10%, 95% would be single microbes.

However, to screen for microbes as rare as 1 in 1,000,000, using this traditional approach would require making at least 10 million droplets, which would in turn require a lengthy FACS process (at 600–700 Hz sorting speed). Therefore, we chose to encapsulate with a Poisson distribution of 1: microbe number = 1*x*, such that ∼37% of drops contain no microbe, ∼37% of drops contain only 1 microbe, 18% of drops contain 2 microbes, 6% of drops contain 3 microbes, and 1.5% of drops contain 4 microbes.

Thus, although some droplets will have more than a single microbe, it will still be sorted out if it contains the target microbe, and a large portion of the reads will map to the target microbe's genome. This tradeoff allowed us to streamline the overall workflow. Based on this distribution, for our test case, we encapsulated ∼3 million microbes, of which three theoretically contain *S. aureus.*

After performing the first step of our workflow, we aligned our sequences to the *S. aureus* reference genome, which provided 1.1% genome coverage (Fig. 2B). For the target enrichment region, we selected an extracellular adherence protein-coding gene that is unique to *S. aureus* and designed a probe and primers specific to this gene. We then used this probe to perform ddPCR in the second droplet split, amplifying the targeted sequence.

We then used single-cell FACS to isolate droplets containing the amplified probe (5 droplets of ∼1.1 million drops). The sorted drops were then subjected to a second round of whole-genome amplification and sequencing (resulting in ∼497 million raw reads). We again aligned the contigs against the reference genome. Approximately 40% of the reads mapped to the *S. aureus* genome. The other reads likely come from a combination of factors, notably the fact that some droplets will have more than just the target microbe encapsulated. It is also possible that some droplets merge during droplet handling, giving rise to off-target sequences.

The assembled contigs covered ∼84% of the *S. aureus* genome with an N50 of 22,001 bp as well as 100% of sequences of the three *S. aureus* plasmids, indicating that although other sequences are present following enrichment, we can achieve high coverage of our target microbe genome (Fig. 2B). Collectively, these contigs covered ∼72% of all *S. aureus* protein-coding genes (Fig. 2C). We were also able to retrieve a 42,903-bp continuous contig containing the target gene of interest, highlighting the power of our method for those interested in studying multigene loci.

**FIG. 3.**
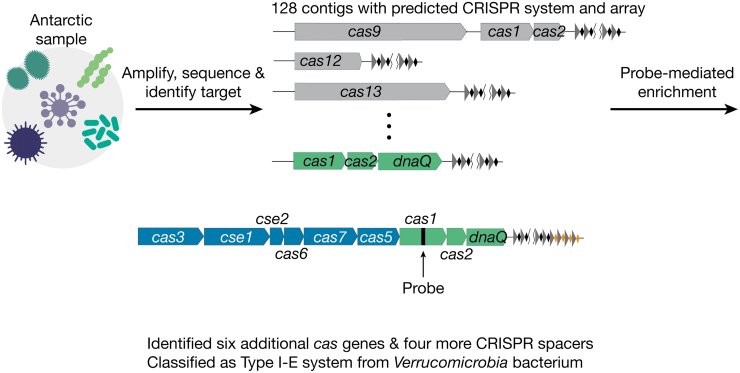
Identification of a previously uncharacterized CRISPR system from an Antarctic metagenomic sample. After performing steps 1 and 2 of the enrichment method, we identified a 2834-bp DNA contig containing *cas1*, *cas2*, *dnaQ*, and 12 CRISPR spacers. We then designed a 208-bp probe targeting *cas1*. After probe-mediated enrichment, we identified six additional *cas* genes as well as four more CRISPR spacers and classified this as a type I-E system from *Verrucomicrobia* bacterium.

We next applied our technology to identify CRISPR systems in a rehydrated, freeze-dried Antarctic lake sample (Fig. 3). After steps 1 and 2 of our enrichment workflow, we identified 128 CRISPR-containing contigs. As a test case, we picked a low-abundance 2834-bp DNA contig containing a CRISPR array, *cas1*, *cas2*, and a gene encoding a protein with a DNA polymerase III (*dnaQ*) exonuclease-like domain.

The presence of a DnaQ domain fused to Cas2 has been reported previously,^23^ but this contig appears to encode a stand-alone DnaQ protein, which may be involved in spacer acquisition.^24^ In addition to this potentially novel gene, we chose this contig as a test case because it contained an incomplete set of CRISPR proteins, precluding determination of the type of system, but did have a CRISPR array, making it likely to be a bona fide system.

Cas1 and Cas2 from this contig share ∼70% protein sequence identity with the closest protein in the NCBI database. However, we could not identify other CRISPR-associated proteins due to the short length of the contig nor the species they belong to because all three proteins matched sequences of *multiple species* at similar levels. We designed a 208-bp probe set targeting the *cas1* gene and used this as a probe to enrich droplets containing this target genome.

After sequencing (∼520 million raw reads) and *de novo* assembly, we were able to retrieve an 8856-bp contig containing six more CRISPR-associated genes (*cas3*, *cse1*, *cse2*, *cas6*, *cas7*, and *cas5*) and four new CRISPR spacers. We performed BLAST searches of the newly identified CRISPR-associated genes and discovered that they all have *Verrucomicrobia bacterium* as the top hit with a 60–80% protein sequence identity. We therefore concluded that this contig is derived from a *Verrucomicrobia* species. Furthermore, we could classify it as a type I-E CRISPR system.

## Discussion

We estimate that our method can provide a near-complete whole-genome sequence for a rare (e.g., 1 in 1,000,000) microbe for less than 2000 USD in under a week. By comparison, at current costs and rates of sequencing, it could take tens of millions of dollars and years before such a rare genome was sequenced from a mixed starting sample. Although our method cannot provide 100% coverage of the target genome, particularly in the case where no reference genome exists and there are nonoverlapping contigs, enrichment of the target microbe's genome in long contigs in the final sequence will still provide a valuable tool for researchers interested in studying multigene loci.

However, we note that in the absence of a reference genome, the enriched sequences will contain a fraction of off-target sequences from other microbes. Even in this case, the dramatic enrichment of on-target sequences will provide a tractable starting point for researchers to empirically test hypotheses. For example, if a protein of interest was used as the probe region, and the researcher is interested in finding interacting partners of that protein within the genome, the enrichment method will suffice to enable experimental testing of a reasonable number of candidates.

In addition to performing affordable and rapid probe-mediated enrichment, which enables enrichment of any known sequence of interest, our method can also be used for enrichment of unknown sequences by depleting droplets containing known sequences and then sequencing the resulting population. Such an approach could enable discovery of novel microbes that have low abundance.

Finally, this method is not limited to microbial samples: it can be applied to mammalian cells as well, enabling studies of rare cells of interest.
